# Radiation Hardness Property of Ultra-Fast 3D-Trench Electrode Silicon Detector on N-Type Substrate

**DOI:** 10.3390/mi12111400

**Published:** 2021-11-14

**Authors:** Manwen Liu, Xinqing Li, Wenzheng Cheng, Zheng Li, Zhihua Li

**Affiliations:** 1 Institute of Microelectronics, Chinese Academy of Sciences (IMECAS), Beijing 100029, China; change0917@163.com (W.C.); 3636@ldu.edu.cn (Z.L.); 2 School of Physics and Optoelectronic Engineering, Ludong University, Yantai 264025, China; xqli1996zz@163.com; 3 School for Optoelectronic Engineering, Zaozhuang University, Zaozhuang 277160, China

**Keywords:** ultra-fast 3D-trench electrode silicon detector, full depletion voltage, breakdown voltage, leakage current, capacitance, weighting field, transient induced current

## Abstract

The radiation fluence of high luminosity LHC (HL-LHC) is predicted up to 1 × 10^16^ 1 MeV n_eq_/cm^2^ in the ATLAS and CMS experiments for the pixel detectors at the innermost layers. The increased radiation leads to the degradation of the detector properties, such as increased leakage current and full depletion voltage, and reduced signals and charge collection efficiency, which means it is necessary to develop the radiation hard semiconductor devices for very high luminosity colliders. In our previous study about ultra-fast 3D-trench electrode silicon detectors, through induced transient current simulation with different minimum ionizing particle (MIP) hitting positions, the ultra-fast response times ranging from 30 ps to 140 ps were verified. In this work, the full depletion voltage, breakdown voltage, leakage current, capacitance, weighting field and MIP induced transient current (signal) of the detector after radiation at different fluences will be simulated and calculated with professional software, namely the finite-element Technology Computer-Aided Design (TCAD) software frameworks. From analysis of the simulation results, one can predict the performance of the detector in heavy radiation environment. The fabrication of pixel detectors will be carried out in CMOS process platform of IMECAS based on ultra-pure high resistivity (up to 10^4^ ohm·cm) silicon material.

## 1. Introduction

The 3D electrode detector was developed by S. Parker et.al in 1997, with N+ and P+ columns etched and doped along the detector thickness [1,2,3]. The full depletion voltage is only depending on the N+ and P+ electrodes spacing, which is insensitive to the detector thickness. In 2011, in order to solve the non-homogeneous electric field distribution and saddle points in the electric potential distribution where low or zero electric field regions exist, Z.Li proposed 3D-Trench electrode silicon detector, which is radiation hard for HL-LHC and X-ray applications [4,5].

With further development of this technology, some variants of the devices have been proposed, such as the single type column (3D-STC) [6], one sided 3D detector [7,8,9], double-side double-type column detector [10,11,12,13,14,15,16], hybird pixel detector [17], ultra-thin 3D silicon detector [18], and the SINTEF 3D active edge silicon detector [19,20], Small-Pitch 3D Pixel detector [21,22]. 3D strip and pad detectors irradiated with neutrons up to a fluence of 3 × 10^17^ n_eq_/cm^2^ are characterised in 2020 [23].

The upgrade of the LHC to its high luminosity (HL-LHC) is scheduled for completion in 2023, radiation fluences exceed 1 × 10^16^ 1 MeV n_eq_/cm^2^ in ATLAS and CMS are predicted for pixel detectors of the innermost layers and more than 10^15^ n_eq_/cm^2^ for the detectors at a radius of 20 cm from vertex [24,25]. In reference [4], the author stated that the electrode spacing of 3D electrode detector can be made on the order of 30 μm to 50 μm, which is close to the trapping distance of free carriers in silicon after high radiation fluence (near 1 × 10^16^ 1 MeV n_eq_/cm^2^) [26], then the carrier trapping can be reduced significantly, resulting in the improvment of the charge collection efficient. There are some works focusing on electrical characteristics of the square 3D-Trench electrode detectors when the electrode spacing is about 50 μm [27,28,29], including electrostatic potential, electric field, leakage current, capacitance, full depletion voltage, and charge collection property. In reference [28], researchers pointed out that the charge collection can still be improved by further reducing the electrode spacing.

In our previous works about the ultra-fast 3D electrode detectors [30,31,32], we predicted the intrinsical radiation hardness for ultra fast 3D trench electrode detectors due to their very small electrode spacing, ranging from 5 μm to 20 μm, which is much smaller than the trapping distance of free carriers in silicon after radiation of 1 × 10^16^ n_eq_/cm^2^. In this work, we increase radiation fluence further and study the degradation of the detector properties, such as increased leakage current and full depletion voltage with professional software. From analysis of the simulation results, one can predict the performance of the detector in heavy radiation environment.

Recent years, radiation damage models, such as the “Perugia Surface” damage modeling scheme [33,34] and the Hamburg Penta Trap Model (HPTM) [35], are proposed to implemented within the “optimiser” TCAD environment. Those models provide the versatility and generality of the simulation methods. Due to these models are put forward in recent years, some are still in the experimental stage, so this work will not consider directly using these models. In addition, the radiation damage model proposed for P-type silicon would not fit this study [36]. In the simulation process, we take the radiation damage effect in reference [37] into consideration while using the classical physical models, such as Shockley-Read-Hall Generation-Recombination. In reference [37], the degradation of silicon sensors/detectors leading by radiation damage has been studied. For example, the increasing of the leakage current, bulk resistivity, space charge concentration and free carrier trapping. When the total fluence is in the order of 1 × 10^15^ n_eq_/cm^2^, the increase in space charge concentration is the main problem, which will increase the full depletion voltage significantly. However, when the total fluence increases up to 1 × 10^16^ n_eq_/cm^2^, the main limiting factor for Si detector operation is the severe trapping of free carriers by radiation-induced defect levels.

In the process of simulation, we define the carrier Mobility model in the Mobility statement, which includes the reduction of electron or hole Mobility due to high Doping Dep and the high electric field (e High Field saturation, h High Field saturation) and reduced mobility (Enormal) at the semiconductor-insulator interface. According to Slotboom model, because of doping, Effective Intrinsic Density (Old Slot boom) activate the narrow band gap. In addition, the corresponding carriers are generated in the recombination (SRH Auger avalanche) activation carrier continuum equation.

## 2. Device Modeling

**Figure 1:**
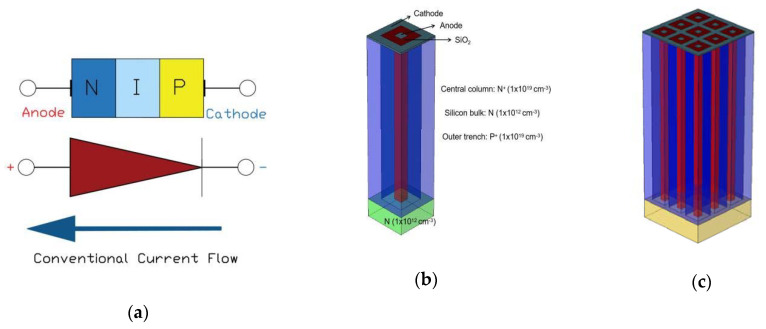
Structure schematic of the square ultra-fast 3D-Trench electrode Si detector; (**a**) Working principle of the detector; (**b**) Unit cell; (**c**) 3 × 3 array.

In this work, a square 3D-Trench electrode silicon detector with electrode spacing ranging from 5 μm to 20 μm is studied by Sentaurus TCAD tool, for detectors irradiated at different fluences. The silicon bulk is N type with a doping concentration of 1 × 10^12^ cm^−3^. The central column electrode is in square shape with 180 μm depth, 5 μm width and N+ doping concentration of 1 × 10^19^ cm^−3^, working as the collection Anode of the detector. The trench electrode is with 180 μm depth, 5 μm width and P+ doping concentration of 1 × 10^19^ cm^−3^ as the detector Cathode. There is a 20 μm thick silicon bulk in the bottom of the device as a support base. The working principle and structure schematic are shown in Figure 1. The structure forms a PIN junction diode. The Anode and Cathode electrodes are separated by a SiO_2_ layer, and its oxide charge density is set as 4 × 10^11^ cm^−2^, which may increase as the radiation increases.

Since the electrode spacing in this study is smaller than 20 μm, the SiO_2_ surface region between electrodes will be very small. Meanwhile, since the trench electrode is P+ doping, we approximately take it as the function of P-stop or P-spray. The difficulty of the 3D electrode detector manufacturing process lies in the etching of trench electrode. At present, Bosch technology can be used to achieve the process of 50:1 aspect ratio, thus providing the possibility of device manufacturing. When connecting the detector device chip to the readout circuit, we can consider the multi-layer metallization in the CMOS process, or the central column electrode is etched through to form a double-sided electrode, or we can look forward to the further development of flip chip bonding (FCB) technology in the future.

The effective doping concentration *N*_eff_ of the silicon bulk can be influenced by irradiation, and it is functioned as:

(1) $$N_{\mathrm{eff}}\approx \beta \Phi_{n}\;\;\;\;(\mathrm{for}\;\Phi_{n}>\;1\times 10^{14\;}n_{\mathrm{eq}}\cdot {\mathrm{cm}}^{-2})$$

where *Φ*_n_ is the 1 MeV neutron-equivalent fluence, the parameter *β* is the introduction rate for deep acceptors, and it is 0.01 here.

## 3. Electrical Characteristics of the Ultra-Fast 3D-Trench Electrode Detector with Variety Radiation Fluences

### 3.1. Leakage Current

In this work, electrons will be collected by the Anode electrode due to the detector bulk is N type silicon and the Anode is N+ doping. When we neglect the surface effect, and assume the recombination centers are in the middle of the energy gap, the leakage current in the depletion region can be presented as [38]:

(2) $$J\approx J_{\mathrm{gen}}=\frac{en_{i}Vol_{\mathrm{dep}}}{2\tau }$$

where *e* is the electronic charge, *n_i_* is the Si intrinsic carrier concentration, and *τ* is the minority carrier lifetime. The leakage current can be affected by the defects that is caused by the radiation, and the defects will increase linearly with radiation fluence, which can be written as [39]:

(3) $$\frac{J}{Vol_{\mathrm{dep}}}=\alpha \Phi_{n}$$

where the damage constant *α* can be chosen as 4 × 10^−^^17^ A/cm [34], and *Vol_dep_* is the depletion volume of the device.

**Figure 2:**
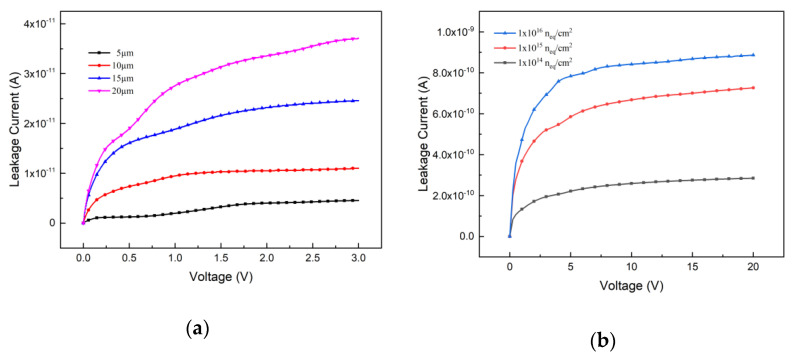
Leakage current curves v.s. bias voltage; (**a**) With the influence of the electrode spacing; (**b**) With the influence of the radiation fluence.

From Equations (2) and (3), one can know that the leakage current can be influenced by the depletion volume and radiation fluence. In Figure 2, the leakage current v.s. bias voltage curves are presented. Since the 3D detector can work at room temperature, the default 300 K in the silicon material parameter file remains unchanged, and the simulation is carried out under constant temperature. From Figure 2a, one can observe that with the increase of the electrode spacing and bias voltage, both corresponding to the increase of depletion volume, the leakage current increases. Figure 2b presents the influence of the radiation fluence to the leakage current. When the electrode spacing is 5 μm, the leakage current increases with the radiation fluence.

### 3.2. Capacitance

**Figure 3:**
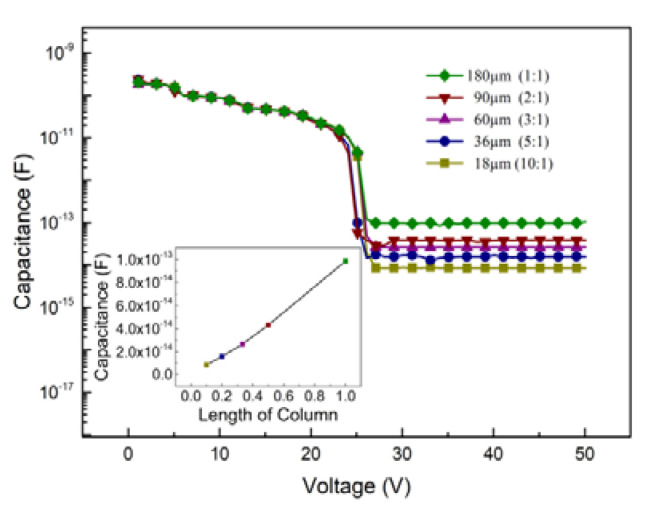
C-V characteristics at different lengths of the column electrode.

From ref. [28], the geometry capacitance of the 3D-trench electrode detector can be approximately expressed as:

(4) Ccyl=2πεrε0dln(Rr)

where *ε_r_ε_0_* is the product of relative and vacuum permittivity, *d* is the length of the column, and *R/r* is the ratio of the outer electrode radii and inner electrode radii of the detector. Figure 3 shows the capacitance v.s. bias voltage curves at different lengths of the column electrode. As we calculated above, the capacitance increases linearly with the column electrode length and they are all less than 100 fF.

### 3.3. The Transient Induced Current

**Figure 4:**
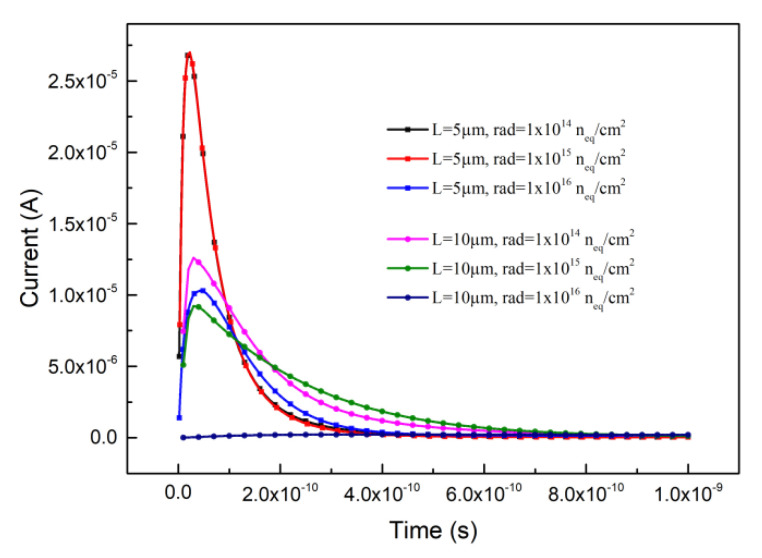
The induced current curves with transient time.

Assume that a MIP (Minimum Ionising Particle) is hitting vertically from the front of the detector, and generate 80 electron-hole pairs/μm along the particle’s track. Then according to Ramo Theorem, the transient induced current can be expressed as:

(5) $$i(t)=q\cdot {\vec{v}}_{d}*{\vec{E}}_{\mathrm{wf}}$$

where the carrier charge *q*=80 e’s/μm×*d_eff_* (*d_eff_* is the effective thickness, which is taken the electrode thickness 180 μm in this work) [28], *v_dr_* is the drift velocity for carriers (electrons or holes), and *E_w_* is the weighting field that will be simulated in this work later. The drift velocity *v_dr_* can be expressed as the functional of the electric field *E(r)* and saturation velocity *v_s_* (1 × 10^7^ cm/s):

(6) $$v_{\mathrm{dr}}=\frac{dr}{dt}=\frac{\mu E(r)}{1+\frac{\mu E(r)}{v_{s}}}$$

where *μ* is the carrier’s low field mobility, and *E* is the electric field in the detector. Current signals are obtained through simulation, as shown in Figure 4. The incident position of MIP was simulated not randomly, but from the middle position of the effective silicon body of the detector. Figure 4 shows that increasing in radiation and electrode spacing will reduce the induced current by affecting the intensity of the electric field [40] and trapping carriers through the defect. In addition, from Figure 4, it is shown that the transient times corresponding to the signal FWHM are ranging from 1 × 10^−10^ s to 3 × 10^−10^ s, namely 100 ps to 300 ps. And current signal peaks are located below 100 ps.

### 3.4. Full Depletion Voltage and Breakdown Voltage

**Figure 5:**
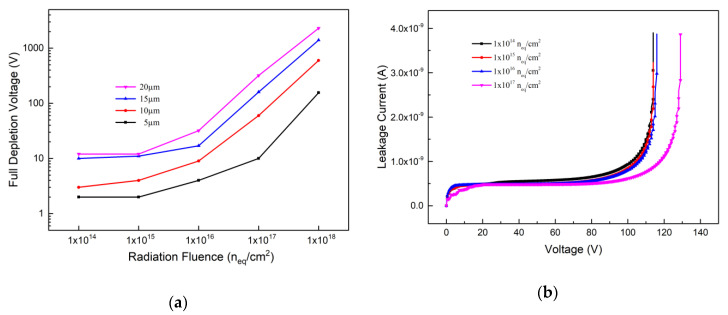
(**a**) The full depletion voltages; (**b**) Breakdown voltages.

The full depletion voltage can be extracted from the C-V and I-V curves. Figure 5a presents the variation trend of full depletion voltage with radiation fluence. The full depletion voltage is only a few volts after radiation with fluence of 1 × 10^14^ n_eq_/cm^2^ while electrode spacing is ranging from 5 μm to 10 μm. When the radiation fluence is increased to 1 × 10^18^ n_eq_/cm^2^, the detector full depletion voltage with electrode spacing of 20 μm is increased up to 1150 V. However, the full depletion voltages with electrode spacing of 5 μm and 10 μm are still below 500 V. Usually, the working bias voltage is larger than the full depletion voltage, which indicates that with very small electrode spacing, even at a very high radiation fluence, bias voltages of several hundred volts can make the device work. In Figure 5b, the abscissa of the inflection point of the curve rising sharply is the breakdown voltage point. From Figure 5b, one can observe that the breakdown voltage will increase with the radiation fluence for the detector with an electrode spacing of 5 μm, and it is about 115 V at radiation fluence of 1 × 10^16^ n_eq_/cm^2^, much larger than the full depletion voltage (about 3 V here).

**Figure 6:**
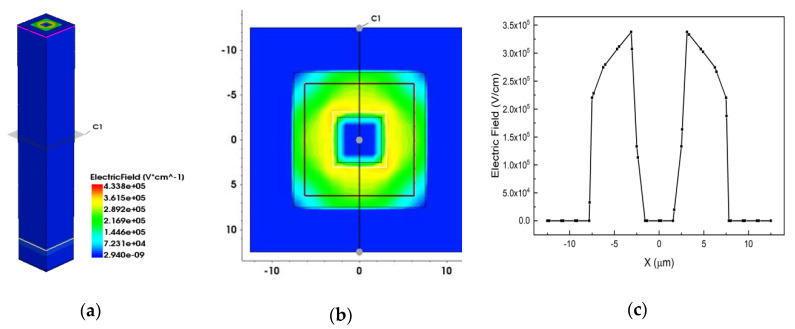
The electric field distribution of the device at the breakdown voltage; (**a**) 3D distribution; (**b**) 2D profile; (**c**) 1D cutline.

Figure 6 show the 3D, 2D and 1D electric field distributions above the breakdown voltage (150 V here) with electrode spacing of 5 μm. Figure 6b is the C1 profile from Figure 6(1). Figure 6c is the C1 cutline from Figure 6(2). From Figure 6, we can observe that the electric field of most detector effective area is larger than 3 × 10^5^ V/cm, which indicates that the detector has been breakdown.

### 3.5. The Weighting Field

From Equations (2) and (3), the minority carrier lifetime can be calculated as:

(7) $$\tau =\frac{en_{i}}{2\alpha \Phi_{n}}$$

The minority carrier lifetime will be influenced by the radiation fluence, and which is reflected by Shockley-Read-Hall (SRH) recombination model during simulation. According to the empirical expression from ref. [41,42], *τ^−^*^1^ = 5 × 10^−7^·*Φ*_n_.

For a detector, the collected charge induced by a MIP in time interval *t* to *t + dt* can be expressed as:

(8) $$dQ=q\cdot v(r)\cdot E_{w}(r)\cdot dt=q\cdot v(r)\cdot E_{w}(r)\cdot \frac{dr}{v(r)}=q\cdot E_{w}(r)\cdot dr$$

**Figure 7:**
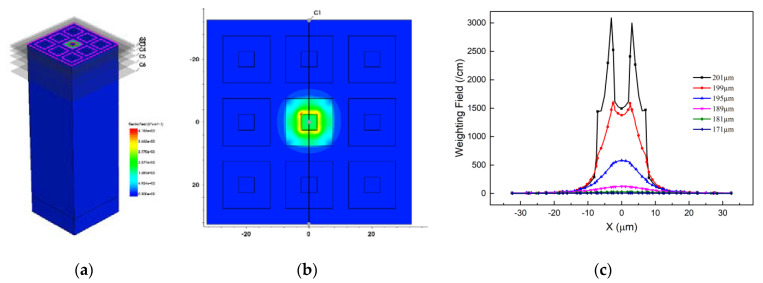
The (**a**) 3D; (**b**) 2D; and (**c**) 1D weighting field distribution.

Which presents that the charge collection of the device is influenced by the weighting fields distribution in the device effective area. Figure 7 presents the weighting field of the 3 × 3 array. From Figure 7c, one can observe that the weighting field decreases as the Z axis decreases, and the trench electrode is acted as a good isolation. Higher specific weighting field values ensures better charge collection of the detector.

## 4. Conclusions

It was predicted that the ultra-fast 3D-silicon detector with trench electrode structure can be ultra radiation hard due to the reduced carrier drift distance. In this work, we verified the prediction through simulation and calculation of leakage current, induced current, capacitance, full depletion voltage, breakdown voltage and the weighting field distribution with different radiation fluences. The leakage current increases with the increasing of the electrode spacing and bias voltage due to fact that they are corresponding to the increase of depletion volume, and it is affected by the radiation fluence. However, increasing in radiation and electrode spacing reduced the induced current by affecting the intensity of the electric field and trapping carriers through the defect. The capacitance of the detector is less than 100 fF when the electrode width is 5 μm, and it increases as the column electrode length increases. The full depletion voltages increase with the electrode spacing and radiation fluences as well, and it is below 500 volts when the electrode spacing is 10 μm even the radiation fluence is up to 1 × 10^18^ n_eq_/cm^2^. The breakdown voltage is larger than 100 volts when the electrode spacing is 5 μm. The weighting field decreases as the Z axis decreases, and the trench electrode is acted as a good isolation.
